# Efficacy analysis of neuroprotective drugs in patients with acute ischemic stroke based on network meta-analysis

**DOI:** 10.3389/fphar.2024.1475021

**Published:** 2024-11-07

**Authors:** Mei Li, Xianhao Huo, Qing Chang, Xiaozhuo Liu, Jianning Zhang, Zhiqi Mao

**Affiliations:** ^1^ Department of Neurosurgery, First Medical Center, Chinese PLA General Hospital, Beijing, China; ^2^ Department of Neurosurgery, General Hospital of Ningxia Medical University, Yinchuan, China; ^3^ Department of Neurosurgery, North China University of Science and Technology Affiliated Hospital, Tangshan, Hebei, China

**Keywords:** ischemic stroke, edaravone, citicoline, ginkgolide, network meta-analysis

## Abstract

**Objective:**

This network meta-analysis aims to explore the efficacy and safety of neuroprotective agents in patients with ischemic stroke and attempts to identify which drug is the most effective in improving outcomes for patients with acute ischemic stroke (AIS) through a ranking method.

**Methods:**

We comprehensively searched the PubMed, Medline, Embase, Web of Science, and Cochrane library databases from their establishment to 30 June 2024. Data were extracted from the studies identified, and their quality was assessed using the Cochrane risk-of-bias tool or the Newcastle–Ottawa Scale (NOS). The outcome measures were for a favorable prognosis, based on the modified Rankin Scale score (mRS) or National Institutes of Health Stroker Scale (NIHSS) score, mortality, and adverse effect with different drug regimens. We utilized Stata version 16.0 and Review Manager (RevMan) version 5.3.0 for statistical analysis.

**Results:**

A total of 35 studies were included: 25 randomized control trials, eight retrospective studies, and two prospective studies. The total sample size was 18,423 cases and included nine interventions: citicoline, edaravone (EDV), edaravone dexborneol, cinepazide maleate, cerebrolysin, minocycline, ginkgolide, ginkgo diterpene lactone meglumine (GDLM), and conventional (CON) treatment. Our analysis revealed that, except for edaravone dexborneol, the ginkgolide, EDV, cinepazide maleate, citicoline, cerebrolysin, minocycline, and GDLM treatment schemes reduced the mortality of patients with AIS compared with CON. Each drug regimen significantly improved the neural function of these patients compared with CON, which from highest to lowest was citicoline + vinpocetine, GDLM, citicoline, edaravone dexborneol, cinepazide maleate, ginkgolide, EDV, and CON. Moreover, we also found that, except for citicoline, the ginkgolide, EDV, edaravone dexborneol, GDLM, and cinepazide maleate treatment schemes had a high total treatment effective rate in these patients, the order from highest to lowest being ginkgolide, EDV, edaravone dexborneol, GDLM, cinepazide maleate, CON, and citicoline. In terms of the ineffective rate, we found that, compared with CON, the edaravone dexborneol, EDV, citicoline, GDLM, ginkgolide, and cinepazide maleate treatment schemes all had a lower ineffective rate. Finally, our analysis revealed that, except for cinepazide maleate and ginkgolide, the EDV, minocycline, edaravone dexborneol, GDLM, citicoline, and cerebrolysin schemes all had a higher rate of adverse effect on patients compared to CON. Based on the impact of the adverse effect with different surgical interventions, we further analyzed the effect of these drug treatments by the total treatment effective rate combined with adverse effect, revealing that EDV, ginkgolide, and edaravone dexborneol were the safest and most effective treatments.

**Conclusion:**

In patients with AIS, ginkgolide, EDV, cinepazide maleate, citicoline, cerebrolysin, minocycline, and GDLM were associated with a reduction in mortality rate. Moreover, ginkgolide, EDV, edaravone dexborneol, and GDLM treatment schemes revealed not only a high total treatment effective rate but also a low rate of treatment inefficacy. When considering the combination of the total treatment effective rate with adverse effect, EDV, ginkgolide, and edaravone dexborneol were revealed as the safest and most effective.

## 1 Background

Ischemic stroke (IS), caused by the interruption of cerebral blood flow due to thrombosis or embolism, is the second leading cause of death globally, with an approximate annual death toll of 5.5 million. It is also a major cause of disability worldwide, with approximately 50% of survivors facing long-term disability (Paul and Candelario-Jalil, 2021). According to epidemiological data from 2017, the worldwide incidence of IS stands at 101.3 cases per 100,000 individuals. Predictions suggest that within the next two decades, the incidence of stroke is expected to rise by approximately 24.9% (Qin et al., 2022; Saini et al., 2021). IS severely affects patients’ quality of life and imposes a heavy economic burden on families and society. Therefore, it is essential to effectively treat this disease.

Current treatment strategies for this disease include, on the one hand, the reperfusion of blood vessels, and on the other hand, the prevention of progressive neuronal injury. Although certain advances have recently been made in the treatment of IS patients through the use of medication and mechanical thrombolysis for revascularization, which has played a positive role in their recovery, the narrow therapeutic time window and the stringent eligibility criteria means that only a minority of patients can truly benefit from such treatment (Hill et al., 2020; Xu et al., 2021). As our understanding of the mechanisms of neuronal injury deepens, an increasing number of researchers are attempting to utilize a variety of neuroprotective techniques to safeguard, restore, or regenerate the functions of the nervous system and its cellular structures (Haupt et al., 2023). To date, hundreds of potential neuroprotective drugs have shown promising evidence in basic and animal experiments but have not demonstrated anticipated therapeutic effects in later clinical trials. In clinical practice, commonly used neuroprotective drugs include edaravone, edaravone dexborneol, citicoline, ginkgo diterpene lactone meglumine, and ginkgolide (Agarwal et al., 2022; Hu et al., 2023; Xu et al., 2019; Zhang et al., 2023; Zhang et al., 2021). However, the choice of these drugs for treatment in clinical work primarily relies on the experience of physicians and individualized decision-making. There is a need for more high-quality clinical trials to verify the efficacy and safety of these neuroprotective drugs in specific patient populations. Moreover, there is insufficient evidence to determine which drug is more suitable for neuroprotective treatment in these patients.

Therefore, our research team conducted a comprehensive and systematic search for all randomized controlled trials and observational studies related to the application of neuroprotective drugs such as edaravone, citicoline, ginkgo diterpene lactone meglumine, and ginkgolide in patients with IS. We aimed to apply network meta-analysis to synthesize both direct and indirect evidence. This study explores the efficacy and safety of these neuroprotective drugs in IS patients from aspects such as neurological function recovery and safety. Additionally, it attempts to identify which drug demonstrates the best effect in improving the prognosis of IS patients by a ranking method.

## 2 Patients and methods

The systematic review and network meta-analysis were performed according to the checklist of the Preferred Reporting Items for Systematic Reviews and Meta-Analyses (PRISMA) extension statement for network meta-analysis.

### 2.1 Inclusion and exclusion criteria

The inclusion and exclusion criteria for this study were based on the PICOS strategy (P: patient/population; I: intervention; C: comparison/control; O: outcome; S: study design). We included patients aged ≥18 years who were diagnosed with first acute ischemic stroke, National Institutes of Health Stroker Scale (NIHSS) > 3, the onset time (from the stroke onset to the began treatment) being ≤72 h. Interventions in the treatment group involved edaravone (EVD), citicoline, ginkgolide, ginkgo diterpene lactone meglumine (GDLM), and so on. Our study design included randomized or non-randomized controlled clinical trials with a focus on studies published in English and ≥10 cases. The outcome indicators were favorable prognosis, which was based on the modified Rankin scale score (mRS) or NIHSS score (markedly effective: mRS = 0, NIHSS >90%; effective: mRS = 1–3, NHISS = 50–90%; ineffective: mRS = 4-5, NIHSS <50%; total treatment effective rate = (marked effective + effective)/n), mortality, and adverse effects.

The exclusion criteria included other types of stroke (e.g., hemorrhagic stroke and transient ischemic attack), patients with severe drug allergy, patients with severe heart, liver, or renal dysfunction, single case reports, single-arm trials without controls, related trials without outcome indicators, animal experiments, duplicate publications, and reviews.

### 2.2 Search strategy

The retrieval formula—(((((ischemic stroke) OR (stroke)) OR (brain stroke)) OR (cerebral stroke)) OR (brain ischemic stroke)) OR (cerebral ischemic stroke)) AND (((edaravone) OR (citicoline)) OR (ginkgolide)) OR (ginkgo diterpene lactone meglumine) —was used to conduct a comprehensive search in the PubMed, Cochrane Library, Embase, Medline, and Web of Science databases from their establishment to 30 June 2024. Relevant references, published systematic reviews, articles included in the meta-analysis, abstracts of conference papers, and ongoing or completed unpublished trials in the World Health Organization clinical registries were searched manually.

### 2.3 Data screening and quality evaluation

The inclusion and exclusion criteria were applied by two reviewers. They independently screened the literature-retrieval results and used the Cochrane quality evaluation method to assess all randomized clinical trials (RCTs) from six aspects: randomization, allocation concealment, blind, selective bias, incomplete data, selective reporting, and other biases. Identified prospective study, retrospective study, case control study, and other non-randomized controlled trials were evaluated by the Newcastle–Ottawa Scale (NOS) from three perspectives: selectivity, comparability, and outcome. Any problems or disagreements encountered during the screening of articles for inclusion in the analysis and quality assessment were resolved by two researchers after consultation. If consensus could not be reached, a third researcher was consulted to provide an objective assessment and facilitate resolution.

### 2.4 Data extraction

These data were extracted from all identified studies: author, publication year, country, study type, age, intervention measures, number of people in each intervention group, details of treatment options, mortality in patients with AIS in different treatment groups, effectiveness of treatment in the different groups, and rates of adverse effects in the different groups. If data were missing, we contacted the corresponding author of the study when possible.

### 2.5 Statistical analysis

The heterogeneity test was conducted for all included studies. When *p* > 0.1 and *I* ^*2*^ < 50%, the results were considered non-heterogeneous, and the fixed-effects model was adopted. Otherwise, the random-effects model was adopted. We used the two-tailed statistical test and considered the difference statistically significant when *p* < 0.05. In the network meta-analysis, we used the surface under the cumulative ranking curve (SUCRA) to rank the treatment effects. In addition, before combining direct and indirect evidence, node-splitting was used to conduct a consistency test to determine whether the two could be combined. The statistical analyses were performed using RevMan 5.3 (Cochrane Collaboration, London, United Kingdom) and Stata 16.0 (StataCorp, TX, United States of America).

## 3 Results

### 3.1 Literature search results

**FIGURE 1:**
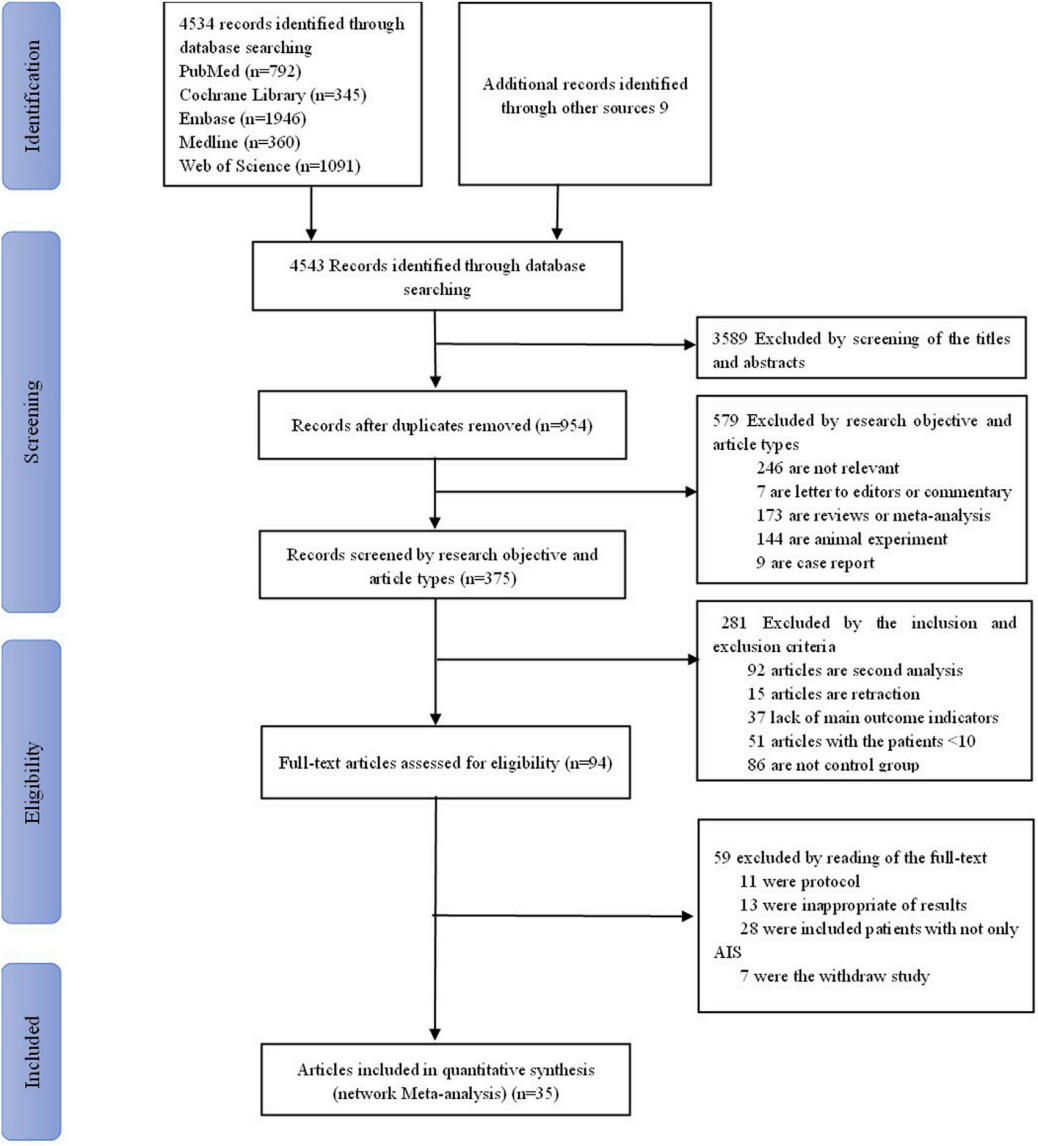
Flow chart of study selection process.

We retrieved 4,543 studies. We removed 3,589 duplicate studies by reading their titles and abstracts. The remaining 954 were screened by reading their research objective and article type, excluding 579 more studies for non-relevance, letters to editors, commentary, reviews, animal experiments, and case reports. In addition, based on inclusion and exclusion, we screened 375 studies and excluded 281 because they were secondary analysis or lacked main outcome indicators. Finally, after excluding a further 59 articles due to protocols, inappropriateness of results, and so on, the remaining 35 were included for network meta-analysis. These were 25 randomized control trials (Xu et al., 2021; Agarwal et al., 2022; Xu et al., 2019; Zhang et al., 2023; Zhang et al., 2021; Alvarez-Sabín et al., 2013; Alvarez-Sabín et al., 2016; Chen et al., 2023; Clark et al., 2001; Clark et al., 1997; Clark et al., 1999; Dávalos et al., 2012; Dong et al., 2021; Goel et al., 2024; Kaste et al., 2013; Li et al., 2020a; Li X.-X. et al., 2020b; Mitta et al., 2012; Ni et al., 2020; Sharma et al., 2011; Shinohara et al., 2009; Sun et al., 2019; Tazaki et al., 1988; Warach et al., 2000; Group, 2003), eight retrospective studies (Hu et al., 2023; Enomoto et al., 2019; Han et al., 2023; Lee and Xiang, 2018; Leon-Jimenez et al., 2010; MIu et al., 2012; Wada et al., 2014; Zhu et al., 2024), and two prospective studies (Mehta et al., 2019; Yamamoto et al., 2011). The screening flow chart is shown in Figure 1. Studies were included from China (n = 14), Japan (n = 6), India (n = 5), the United States of America (n = 5), Spain (n = 2), Russia (n = 1), Mexico (n = 1), and Finland (n = 1). Their study design, publication year, types of interventions, and more other details are shown in Table 1: Supplementary Table S1. The total sample size of 18,423 cases included nine interventions (citicoline, edaravone (EDV), edaravone dexborneol, cinepazide maleate, cerebrolysin, minocycline, ginkgolide, ginkgo diterpene lactone meglumine (GDLM), and conventional treatment (CON)). In addition, all patients in this study were given appropriate secondary prevention interventions, including therapeutic strategies for anti-platelet agents or anti-thrombotic management, lipid reduction, regulation of blood pressure, and control of blood glucose levels.

**TABLE 1:** Baseline characteristics of involved patients.

Study	Country	Publication year	Age(y)	Male	Cases	Outcomes	Follow-up time
Yamamoto et al., 2011	Japan	2011	71.3 ± 11.1/73.0 ± 9.9	66/61	100/118	Death, effective effect/	90 days
Dávalos et al., 2012	Spain	2012	72.9 ± 11.8/72.8 ± 12.1	588/554	1,148/1,150	Death, effective effect, and adverse effect.	90 days
Shinohara et al., 2009	Japan	2009	68.4 ± 11.0/69.1 ± 10.8	121/118	191/195	Death, effective effect, and adverse effect.	90 days
Wada et al., 2014	Japan	2014	66-81/71-85	196/202	356/356	Death, effective effect.	NR
Kaste et al., 2013	Finland	2013	41-77/40-79	19/8	25/11	Death, effective effect, and adverse effect.	90 days
Alvarez-Sabín et al., 2013	United States of America	2013	66.9 ± 11.1/67.7 ± 11.6	92/94	172/175	Death, effective effect, and adverse effect.	6 months
Lee and Xiang, 2018	China	2018	71.9 ± 11.15/74.05 ± 16.21	70/70	132/136	Death, effective effect, and adverse effect.	NR
Clark et al., 2001	United States of America	2001	68/67	50/54	453/446	Death, effective effect, and adverse effect.	90 days
Li et al., 2020a	China	2020	60.5 ± 7.6/62.5 ± 7.9	25/29	48/48	Adverse effect.	NR
Sharma et al., 2011	India	2011	58.12 ± 10.79/56.0 ± 8.15	16/15	25/25	Death, effective effect, and adverse effect.	90 days
Warach et al., 2000	United States of America	2000	68.5/72.1	23/19	41/40	Death, effective effect, and adverse effect.	90 days
Goel et al., 2024	India	2024	60.2/60.2	20/21	30/30	Effective effect.	90 days
Clark et al., 1999	United States of America	1999	70/71	123/62	267/127	Death, effective effect, and adverse effect.	90 days
Mitta et al., 2012	India	2012	57.36 ± 14.79/54.83 ± 14.46/55.6 ± 14.59	13/13/16	22/24/25	Death, effective effect.	90 days
Enomoto et al., 2019	Japan	2019	72-85/72-85	676/677	1,226/1,226	Death, effective effect, and adverse effect.	NR
Alvarez-Sabín et al., 2016	Spain	2016	68.5 ± 9.8/66.4 ± 11.4	39/41	86/87	Effective effect.	2 years
MIu et al., 2012	Russia	2012	Male 64.5 ± 11.5; Female 61.0 ± 12.8	Total 65	89/52	Death, effective effect.	NR
Clark et al., 1997	United States of America	1997	70/67	92/29	194/65	Death, effective effect.	90 days
Li et al., 2020b	China	2020	54.87 ± 7.82/54.08 ± 8.06	40/41	59/59	Death, effective effect.	90 days
Leon-Jimenez et al., 2010	Mexico	2010	68.6/69.6	42/41	86/87	Death, effective effect.	90 days
Sun et al., 2019	China	2019	52.4 ± 4.1/51.3 ± 4.2	37/40	65/65	Death, effective effect, and adverse effect.	90 days
Eiichi O (Group, 2003)	Japan	2003	66.3 ± 8.0/66.1 ± 8.5	82/84	125/125	Death, effective effect, and adverse effect.	90 days
Ni et al., 2020	China	2020	60.3 ± 10.31/62.1 ± 9.65	312/309	466/471	Death, effective effect, and adverse effect.	90 days
Tazaki et al., 1988	Japan	1988	29-90/29-90	87/93	131/136	Death, effective effect, and adverse effect.	NR
Mehta et al., 2019	India	2019	59.5/57.3/58.8/61.9/64.9	12/11/13/12/11	20/20/20/20/20	Death, adverse effect.	90 days
Agarwal et al., 2022	India	2022	61 ± 14.5/54.5 ± 14.6	30/30	49/40	Death, effective effect, and adverse effect.	90 days
Xu et al., 2021	China	2021	62.96/62.86	404/407	599/595	Death, effective effect, and adverse effect.	90 days
Xu et al., 2019	China	2019	35-75/35-75	196/65	291/94	Death, effective effect, and adverse effect.	90 days
Zhu et al., 2024	China	2024	66.26 ± 4.97/66.14 ± 4.85	31/29	57/56	Death, effective effect, and adverse effect.	90 days
Dong et al., 2021	China	2021	64.31 ± 10.68/64.12 ± 10.40	283/316	463/473	Death, effective effect, and adverse effect.	90 days
Han et al., 2023	China	2023	66.4 ± 11.3/66.5 ± 10.4	108/109	161/161	Effective effect, adverse effect.	90 days
Zhang et al., 2021	China	2021	68 ± 12/68 ± 12	152/164	404/404	Death, effective effect, and adverse effect.	90 days
Chen et al., 2023	China	2023	70.40 ± 9.88/70.97 ± 11.19	21/29	35/35	Effective effect, adverse effect.	90 days
Zhang et al., 2023	China	2023	63/63	1,085/1,131	1725/1723	Death, effective effect, and adverse effect.	90 days
Hu et al., 2023	China	2023	65.8 ± 11.0/66.6 ± 12.7	45/44	69/73	Death, effective effect.	90 days

^a^ EDV: edaravone; NR: not report.

### 3.2 Quality evaluation

**FIGURE 2:**
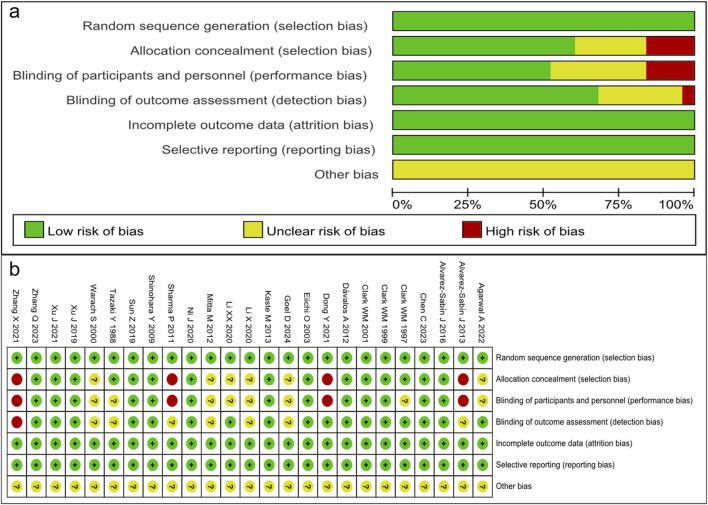
Quality assessment of identified randomized controlled trials. **(A)** Each risk of bias item presented as percentages across all included studies. **(B)** Each risk of bias item for each included study. Green indicates a low risk of bias, yellow an unclear risk of bias, and red a high risk of bias.

The 25 randomized trials identified were assessed by using the Cochrane risk-of-bias tool using the correct randomization method and having complete data. Except for Alvarez-Sabín et al. (2013), Sharma et al. (2011), Dong et al. (2021), and Zhang et al. (2021), they did not use allocation concealment and blinding of participants and personnel. Moreover, it is unclear whether implementation of the allocation concealment and blinding was performed correctly in Agarwal et al. (2022), Goel et al. (2024), Li et al. (2020a), Li et al. (2020b), Mitta et al. (2012), Warach et al. (2000). Thus, the quality of the RCTs included in the analysis was moderate (Figures 2A, B). The NOS assessment tool was used for the other retrospective and prospective studies, which scored highly in selectivity, comparability, and results, indicating that the retrospective and prospective studies identified were of high quality (Table 2).

**TABLE 2:** Quality assessment of non-RCT studies.

Study	Selection	Comparability	Outcome	Total score
Yamamoto Y	4	2	2	8
Wada T	4	2	2	8
Lee XR	4	2	3	9
Enomoto M	4	1	3	8
Martynov Mlu	4	2	2	8
Leon-Jimenez C	4	2	3	9
Mehta A	4	1	3	9
Zhu X	4	1	2	7
Han X	4	2	3	9
Hu X	4	2	1	7

^a^ score of 5 or less indicates high risk of bias.

### 3.3 Traditional meta-analysis

In the subgroup analysis in terms of mortality in patients with AIS after the treatment of neuroprotective drugs, there was a heterogeneity between the subgroups (*I* ^*2*^ > 50%, *p* < 0.1), so the random-effects model was adopted (Supplementary Figure S1A). The analysis showed that EDA and ginkgolide treatment schemes significantly reduced mortality in patients with AIS compared to the CON treatment, with a statistically significant difference (all *p* < 0.00001). However, compared with placebo treatment, citicoline, cinepazide maleate, GDLM, and edaravone dexborneol did not reduce mortality in patients with AIS, and the differences were not statistically significant (all *p* > 0.05). Moreover, compared with EDV treatment, citicoline and edaravone dexborneol also did not reduce mortality, with no statistical differences (all *p* > 0.05).

The subgroup analysis in terms of the proportion of patients with AIS having favorable outcomes and the total treatment effective rate after these drug treatments revealed significant heterogeneity among the subgroups (I^2^>50%, *p* < 0.1), so a random-effect model was adopted (Supplementary Figures S1B, C). In terms of favorable outcomes, the study revealed that the EDV, citicoline, citicoline + vinpocetine, cinepazide maleate, and GDLM treatment schemes significantly improved the neural function with AIS patients compared with CON treatment, with statistically significant differences (all *p* < 0.05). However, compared with CON treatment, ginkgolide and edaravone dexborneol did not significantly improve the neural function of patients with AIS, and the differences were not statistically significant (*p* = 0.20 and *p* = 0.23). Moreover, compared with EDV, citicoline and edaravone dexborneol also did not significantly improve the neural function of patients with AIS, and the differences were not statistically significant (*p* = 0.56 and *p* = 0.08). In terms of the total treatment effective rate, the study revealed that EDV and ginkgolide treatment schemes had a high total treatment effective rate with these patients compared with CON, with statistically significant differences (all *p* < 0.05). In addition, other subgroups were not significantly different in total treatment effective rate (all *p* > 0.05).

The subgroup analysis in terms of the proportion of patients with AIS having adverse effect after drug treatment revealed no heterogeneity among the subgroups (*I* ^*2*^ < 50%, *p* > 0.1), so the fixed-effect model was adopted (Supplementary Figure S1D). The study revealed that, compared with CON, the rate of adverse effect after these drug treatments was not significantly increased by EDV, citicoline, cinepazide maleate, ginkgolide, and GDLM . Moreover, the study also revealed that the citicoline and edaravone dexborneol did not increase the rate of adverse effect of patients with AIS compared with EDV, with no significant statistical difference.

### 3.4 Network meta-analysis

#### 3.4.1 Network diagram of different intervention measures

**FIGURE 3:**
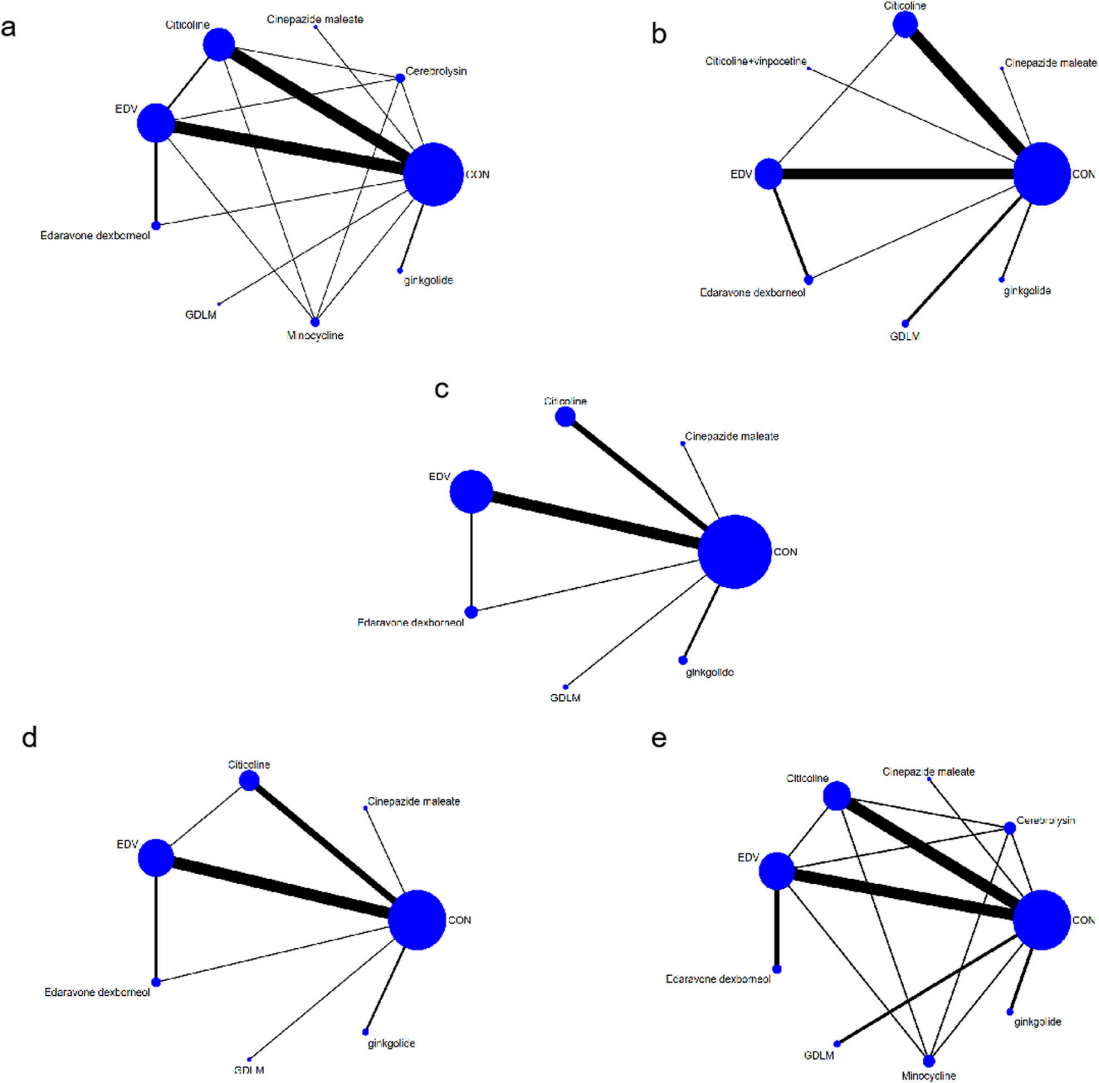
Network chart **(A)** based on the mortality of AIS; **(B)** based on the patient proportion of the favorable result of AIS; **(C)** based on the patient proportion of the total treatment effective rate of AIS; **(D)** based on the patient proportion of the ineffective rate of AIS; **(E)** based on the patient proportion of the adverse effect of AIS.

A direct comparison is shown if there is a direct line between the two intervention groups, but if there is no line, there is no evidence of a direct comparison. The size of dots in the figure represents the sample size, and the thickness of lines represents the number of studies. The drug treatment schemes were indirectly compared using CON as a medium, with ten interventions: EDV, citicoline, edaravone dexborneol, ginkgolide, GDLM, minocycline, cerebrolysin, cinepazide maleate, citicoline + vinpocetine, and CON (Figures 3A–E).

#### 3.4.2 Inconsistency test

There was no direct or indirect comparative evidence in the included studies, so no inconsistency test was conducted (Supplementary Figures S2A–E).

#### 3.4.3 Sequence diagram of network meta-analysis

**FIGURE 4:**
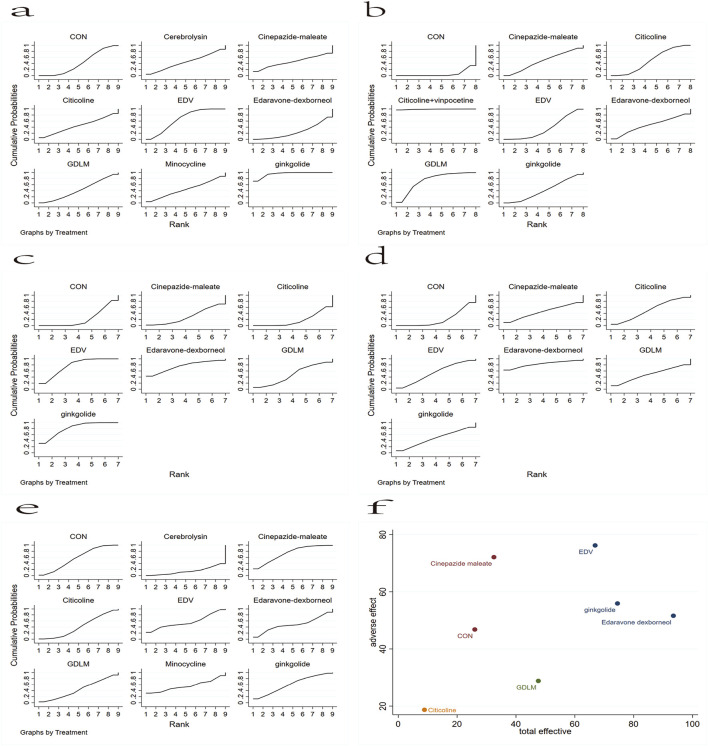
Rank chart: **(A)** based on the mortality of AIS; **(B)** based on the patient proportion of the favorable result of AIS; **(C)** based on the patient proportion of the total treatment effective rate of AIS; **(D)** based on the patient proportion of the ineffective rate of AIS; **(E)** based on the patient proportion of the adverse effect of AIS. **(F)** The rank chart based on the effective effect combined with adverse effect.

The analysis of the mortality of patients with AIS after different drug treatments involved nine different neuroprotective drugs (Figure 4A). In the larger area under the curve, these drugs have a lower mortality. The analysis revealed that, except for edaravone dexborneol, the mortality of patients with AIS reduced with ginkgolide, EDV, cinepazide maleate, citicoline, cerebrolysin, minocycline, and GDLM compared with CON. The mortality rates ranked from lowest to the highest were ginkgolide, EDV, cinepazide maleate, citicoline, cerebrolysin, minocycline, GDLM, CON, and edaravone dexborneol. Analysis in terms of the proportion of patients with AIS who improved neural function (Figure 4B) revealed that each drug treatment intervention significantly improved neural function compared with CON, with the order from highest to the lowest being citicoline + vinpocetine, GDLM, citicoline, edaravone dexborneol, cinepazide maleate, ginkgolide, EDV, and CON.

Subsequently, based on the classification of mRS levels after these drug treatments, we analyzed the total treatment effective and ineffective rates. Figure 4C shows the network meta-analysis sequence diagram for the patient proportion of the total treatment effective rate of AIS. This revealed that, except for citicoline, a high total treatment effective rate of patients with AIS occurred with ginkgolide, EDV, edaravone dexborneol, GDLM, and cinepazide maleate treatment schemes compared with CON. The order from highest to lowest was ginkgolide, EDV, edaravone dexborneol, GDLM, cinepazide maleate, CON, and citicoline. Figure 4D shows the network meta-analysis sequence diagram for the patient proportion of the ineffective rate of AIS. In the larger area under the curve, this drug treatment regimen has a lower proportion of ineffective rate. Thus, compared with CON, the edaravone dexborneol, EDV, citicoline, GDLM, ginkgolide, and cinepazide maleate treatment schemes all had a lower ineffective rate. The order from lowest to highest was edaravone dexborneol, EDV, citicoline, GDLM, ginkgolide, cinepazide maleate, and CON.

We also analyzed the rate of adverse effect of patients with AIS (Figure 4E). In the larger area under the curve, this drug treatment regimen has a lower proportion of adverse effect. This revealed that, except for cinepazide maleate and ginkgolide, the EDV, minocycline, edaravone dexborneol, GDLM, citicoline, and cerebrolysin schemes all had a higher proportion of low adverse effect in these patients compared with CON. The order from lowest to highest was cinepazide maleate, ginkgolide, CON, EDV, minocycline, edaravone dexborneol, GDLM, citicoline, and cerebrolysin. Finally, based on the impact of the adverse effect with different surgical interventions (Figure 4F), we further analyzed these drug treatment effects by the total treatment effective rate combined with adverse effect, revealing that EDV, ginkgolide, and edaravone dexborneol were the safest and most effective.

## 4 Discussion

Currently, thrombolysis stands as the clinical gold standard for treating AIS; however, the brief therapeutic window limits its applicability, resulting in a still relatively constrained range of treatment options available for these patients (Li et al., 2024). A series of cascade reactions are triggered following cerebral ischemia, including inflammatory responses in neurons, apoptosis, and excitotoxicity induced by glutamate (Wu et al., 2020). Furthermore, reperfusion after ischemic injury can lead to additional damage. However, with timely and appropriate interventions, neuronal damage in the peri-ischemic penumbra can be reversed within a short period (Xu et al., 2021). Consequently, an increasing number of neuroprotective drugs are being developed to prevent and treat various types of secondary brain injury (Ren et al., 2023). Unfortunately, no country has currently recommended the use of neuroprotective agents as a standard clinical treatment protocol. In China, commonly utilized neuroprotective agents include edaravone, edaravone dexborneol, GDLM, citicoline, and ginkgolide. The application of these medications predominantly relies on the clinical experience of physicians and personalized treatment strategies, with inadequate scientific evidence to substantiate which drug may be more suitable for such patients.

Based on this, the current study initially conducted a traditional meta-analysis to evaluate the mortality rates, neurological function improvement, and side effects in AIS patients treated with various neuroprotective agents. The analysis revealed that, compared to CON treatment, the EDV and ginkgolide treatment regimens significantly enhanced the treatment effectiveness rate in AIS patients, reduced mortality, and did not increase the incidence of adverse events. The results of this study are comparable to those of Group (2003), Sun et al. (2019), Dong et al. (2021), and Zhang et al. (2021). In the first two studies, it was demonstrated that edaravone can significantly improve the neurological deficits in patients with AIS with a high degree of safety; in the latter two, ginkgolide was also confirmed as offering a safe and effective therapeutic alternative for these patients. Based on the outcomes of this study, we can further corroborate the efficacy of neuroprotective agents (EDV and ginkgolide) in the treatment of patients with AIS. Furthermore, the subgroup analysis of this study indicates that, compared to the CON regimen, the citicoline treatment protocol significantly ameliorates the neurological function of patients with AIS and demonstrates good safety. These findings align with the results of traditional meta-analysis conducted by Secades et al. (2016), which showed that the citicoline treatment protocol is superior to CON in improving the neurological function of such patients. However, that study was confined to randomized controlled trials and did not include a comprehensive summary analysis of the safety profile of the drug therapy. There was also a lack of standardization in the dosages of citicoline, and the impact of different dosages on the drug’s therapeutic efficacy—whether they reduce or enhance it—is uncertain. Therefore, this is an interesting research direction, and it is essential to determine the standard dosage in future studies. In addition, the subgroup analysis revealed that there are no significant differences in mortality rates, neurological improvements, and adverse effects among patients with AIS when comparing neuroprotectant EDV with both the edaravone dexborneol and citicoline regimens. In existing studies that compared EDV with edaravone dexborneol, Xu et al. (2019) arrived at similar conclusions, indicating that edaravone dexborneol did not lead to significant neurological improvements but exhibited good patient tolerability. However, Zhu et al. (2024) and Xu et al. (2021) indicated that, compared with the EDV scheme, the edaravone dexborneol regimen not only significantly enhances neurological recovery in patients with AIS but also does not exhibit a significant increase in adverse effects. Given the contradictory findings, we believe that further research is warranted to determine whether the edaravone dexborneol regimen can replace EDV as a neuroprotective treatment for patients with AIS. Furthermore, existing studies comparing the therapeutic effects of EDV and citicoline (Mitta et al., 2012; Mehta et al., 2019) have also yielded similar conclusions to this study, and there is still currently insufficient evidence to confirm which of the EDV and citicoline regimens is more suitable for such patients.

Based on the subgroup analysis, we found that there was significant heterogeneity in terms of mortality, favorable outcomes, and the total treatment effective rate. Regarding mortality, the reason for heterogeneity on the subgroup of edaravone dexborneol and EDV was mainly attributed to differences in drug dosage. Regarding favorable outcomes, the reason for heterogeneity in the citicoline and CON subgroup was mainly attributed to differences in drug dosage and drug-delivery methods; the reason for heterogeneity in the ginkgolide and CON subgroup was attributed to Zhang et al. (2021) giving thrombolytic therapy before the administration of ginkgolide. Regarding the total treatment effective rate, the reason for heterogeneity in the subgroup of EDV and CON, edaravone dexhoneol, and EDV were both attributed to the differences in study types; Enomoto et al. (2019), Lee and Xiang (2018), and Zhu et al. (2024) are all retrospective studies, while the others are RCTs. The reason for heterogeneity in the ginkgolide and CON subgroup was also attributed to Zhang et al. (2021) giving thrombolytic therapy before the administration of ginkgolide. The presence of heterogeneity may reduce the strength of the evidence in our study. This further highlights the importance of exploring dosage and drug-delivery methods in future research, and also highlights the need for a large number of high-quality, large RCTs to validate our findings.

Network meta-analysis is an extension of traditional meta-analysis, which integrates direct and indirect evidence to compare interventions that were not directly compared in the original trials; it can identify the optimal treatment schemes by ranking methods (Nino and Brignardello-Petersen, 2023; Rouse et al., 2017). In this study, we applied network meta-analysis for the first time to synthesize existing direct and indirect comparative evidence on neuroprotective agents for patients with AIS. The analysis revealed that, in terms of mortality reduction, except for edaravone dexborneol, treatment schemes such as ginkgolide, EDV, cinepazide maleate, citicoline, cerebrolysin, minocycline, and GDLM significantly reduced the mortality of AIS patients compared to CON, with ginkgolide showing the most pronounced effect. In terms of neurological improvement, all neuroprotective agents included in the analysis—citicoline + vinpocetine, GDLM, citicoline, edaravone dexborneol, cinepazide maleate, ginkgolide, and EDV—demonstrated good efficacy in improving neurological function, with citicoline combined with vinpocetine, GDLM, and citicoline showing the best improvement effects. However, given that only one study on the treatment of citicoline + vinpocetine was included in this analysis and the sample size was limited, we cannot conclusively determine the efficacy of this combined treatment on neurological function improvement. Therefore, further clinical research is warranted to ascertain whether this combined therapy has a significant effect on neurological function.

Additionally, we conducted a ranking analysis of the neuroprotective agents included in the study regarding their total effective and ineffective treatment rates. We found that the ginkgolide, EDV, edaravone dexborneol, and GDLM regimens all had a higher total treatment effective rate and a lower ineffective rate. However, the citicoline regimen’s performance in terms of total treatment effective rate was suboptimal. It is well known that the presence of adverse effects can restrict the clinical application of medications. Therefore, we also analyzed the incidence of side effects of these neuroprotective agents by a ranking method and found that EDV, edaravone dexborneol, GDLM, and citicoline all had a higher incidence of adverse effects. Finally, to mitigate the potential for side effects diminishing the therapeutic efficacy of the drugs, we conducted a combined analysis of the total treatment effective rate and the incidence of adverse effects. Our findings revealed that EDV, ginkgolide, and edaravone dexborneol were among the safest and most effective treatments. However, to ascertain which of these three is the most optimal, future high-quality, large-scale, multicenter randomized controlled trials will be necessary for in-depth exploration and analysis.

This study has certain limitations. First, the citicoline and edaravone dexborneol included in this analysis were applied at slightly different doses across various studies, and we did not conduct a detailed analysis based on these different doses, which may have affected the accuracy of the drug’s assessment. Second, there were two drug-delivery methods for citicoline (intravenous and oral), and whether these differences affected treatment efficacy is still uncertain; therefore, this question also should be addressed in future research. Third, this study was limited to literature published in English, potentially leading to omissions due to language restrictions. Additionally, when enrolling AIS patients, the study did not strictly differentiate whether they had received thrombolysis/anticoagulation therapy before or concurrently with neuroprotective agent treatment, making it unclear how thrombolysis/anticoagulation therapy might affect the therapeutic effects of neuroprotective agents. Lastly, the analysis of neuroprotective agents such as minocycline, cerebrolysin, cinepazide maleate, and citicoline + vinpocetine in this study was based on single studies with small sample sizes; this introduces a degree of uncertainty regarding the efficacy and safety conclusions concerning these drugs within the study; further analysis with increased sample sizes is needed in the future.

## 5 Conclusion

This study of patients with AIS revealed that, with the exception of edaravone dexborneol, all other treatment schemes (ginkgolide, EDV, cinepazide maleate, citicoline, cerebrolysin, minocycline, and GDLM) were associated with a reduction in mortality rate. Moreover, the ginkgolide, EDV, edaravone dexborneol, and GDLM treatment schemes revealed not only a high total treatment effective rate but also a low rate of treatment inefficacy. Consideration of the combination of the total treatment effective rate with adverse effect revealed that EDV, ginkgolide, and edaravone dexborneol were the safest and most effective. However, based on the current state of research, we think that there is still need for additional prospective, multicenter studies with long-term follow-up to validate our findings.

## Data Availability

The original contributions presented in the study are included in the article/Supplementary Material; further inquiries can be directed to the corresponding author.
