# Supplementary material for: Efficacy analysis of neuroprotective drugs in patients with acute ischemic stroke based on network meta-analysis
Source: Front Pharmacol. 2024 Nov 7;15:1475021. doi: 10.3389/fphar.2024.1475021 (PMC11578817; doi:10.3389/fphar.2024.1475021)
Supplement: Supplementary file 1 [file Table1.docx]

Supplementary table 1. Overview of the included literature

| Study | Publication year | Study design | Study Type | Intervention  Control | Time from stroke onset to treatment | mRS | NIHSS | Treatment details |
| --- | --- | --- | --- | --- | --- | --- | --- | --- |
| Yamamoto Y^[40]^ | 2011 | Prospective  study | Single institution | EDV  CON | Within 24h  Within 24h | NR  NR | 4.4±2.6  4.1±1.4 | EDV: 30mg was given intravenously, twice daily;  Conventional treatment: comprised oxygen therapy, sedation, antiplatelet aggregation, and other treatment measures that decrease blood pressure and intracranial pressure, promote brain cell metabolism and blood circulation to remove blood stasis, and manage water and electrolyte imbalance. |
| Dávalos A^[18]^ | 2012 | Randomized  clinical trial | Multicenter | Citicoline  CON | 4.0-12.3 h  4.0-12.3 h | NR  NR | 11-19  11-19 | Citicoline 2000mg every day;  Conventional treatment: comprised oxygen therapy, sedation, antiplatelet aggregation, and other treatment measures that decrease blood pressure and intracranial pressure, promote brain cell metabolism and blood circulation to remove blood stasis, and manage water and electrolyte imbalance. |
| Shinohara Y^[27]^ | 2009 | Randomized  clinical trial | Multicenter | EDV  CON | Within 24h  Within 24h | NR  NR | 3.7±2.3  3.8±2.7 | EDV: 30mg was given intravenously, twice daily;  Conventional treatment: comprised oxygen therapy, sedation, antiplatelet aggregation, and other treatment measures that decrease blood pressure and intracranial pressure, promote brain cell metabolism and blood circulation to remove blood stasis, and manage water and electrolyte imbalance. |
| Wada T^[37]^ | 2014 | Retrospective  study | Multicenter | EDV  CON | Within 24h  Within 24h | 0-5  0-5 | NR  NR | EDV: 30mg was given intravenously, twice daily;  Conventional treatment: comprised oxygen therapy, sedation, antiplatelet aggregation, and other treatment measures that decrease blood pressure and intracranial pressure, promote brain cell metabolism and blood circulation to remove blood stasis, and manage water and electrolyte imbalance. |
| Kaste M^[21]^ | 2013 | Randomized  Clinical trial | Multicenter | EDV  CON | Within 72h  Within 72h | NR  NR | 3-9  4-12 | EDV: 30mg was given intravenously, twice daily;  Conventional treatment: comprised oxygen therapy, sedation, antiplatelet aggregation, and other treatment measures that decrease blood pressure and intracranial pressure, promote brain cell metabolism and blood circulation to remove blood stasis, and manage water and electrolyte imbalance. |
| Alvarez-Sabín J^[12]^ | 2013 | Randomized  Clinical trail | Single institution | Citicoline  CON | Within 24h  Within 24h | NR  NR | 10-17  10-16 | Citicoline 1000 mg every day;  Conventional treatment: comprised oxygen therapy, sedation, antiplatelet aggregation, and other treatment measures that decrease blood pressure and intracranial pressure, promote brain cell metabolism and blood circulation to remove blood stasis, and manage water and electrolyte imbalance. |
| Lee XR^[34]^ | 2018 | Retrospective  study | Single institution | EDV  CON | Within 24h  Within 24h | NR  NR | 20.88±8.31  20.67±10.06 | EDV: 30mg was given intravenously, twice daily;  Conventional treatment: comprised oxygen therapy, sedation, antiplatelet aggregation, and other treatment measures that decrease blood pressure and intracranial pressure, promote brain cell metabolism and blood circulation to remove blood stasis, and manage water and electrolyte imbalance. |
| Clark WM^[15]^ | 2001 | Randomized  clinical trial | Multicenter | Citicoline  CON | 13.2h  13.3h | NR  NR | 13.9  14.5 | Citicoline 2000 mg every day;  Conventional treatment: comprised oxygen therapy, sedation, antiplatelet aggregation, and other treatment measures that decrease blood pressure and intracranial pressure, promote brain cell metabolism and blood circulation to remove blood stasis, and manage water and electrolyte imbalance. |
| Li X^[22]^ | 2020 | Randomized  Clinical trial | Single institution | EDV  CON | Within 24h  Within 24h | NR  NR | 26.06±5.23  27.08±5.68 | EDV: 30mg was given intravenously, twice daily; conventional treatment: comprised oxygen therapy, sedation, antiplatelet aggregation, and other treatment measures that decrease blood pressure and intracranial pressure, promote brain cell metabolism and blood circulation to remove blood stasis, and manage water and electrolyte imbalance. |
| Sharma P^[26]^ | 2011 | Randomized  Clinical trial | Single institution | EDV  CON | 30.08±18.87h  25.48±13.59h | 3.84±1.17  4.0±1.08 | 10.56±5.74  10.08±5.66 | EDV: 30mg was given intravenously, twice daily;  Conventional treatment: comprised oxygen therapy, sedation, antiplatelet aggregation, and other treatment measures that decrease blood pressure and intracranial pressure, promote brain cell metabolism and blood circulation to remove blood stasis, and manage water and electrolyte imbalance. |
| Warach S^[30]^ | 2000 | Randomized  Clinical trial | Multicenter | Citicoline  CON | 15.1±0.9h  13.5±1.0h | NR  NR | 11.5±0.7  12.7±0.9 | Citicoline 500 mg every day;  Conventional treatment: comprised oxygen therapy, sedation, antiplatelet aggregation, and other treatment measures that decrease blood pressure and intracranial pressure, promote brain cell metabolism and blood circulation to remove blood stasis, and manage water and electrolyte imbalance. |
| Goel D^[20]^ | 2024 | Randomized  Clinical trial | Single institution | Citicoline+vinpocetine  CON | Within 72h  Within 72h | NR  NR | ≥6  ≥6 | Citicoline 1000mg every day, vinpocetine 10mg daily;  Conventional treatment: comprised oxygen therapy, sedation, antiplatelet aggregation, and other treatment measures that decrease blood pressure and intracranial pressure, promote brain cell metabolism and blood circulation to remove blood stasis, and manage water and electrolyte imbalance. |
| Clark WM^[17]^ | 1999 | Randomized  Clinical trial | Multicenter | Citicoline  CON | 11.7/12.3h | NR  NR | 13.3/12.7 | Citicoline 500 mg every day;  Conventional treatment: comprised oxygen therapy, sedation, antiplatelet aggregation, and other treatment measures that decrease blood pressure and intracranial pressure, promote brain cell metabolism and blood circulation to remove blood stasis, and manage water and electrolyte imbalance. |
| Mitta M^[24]^ | 2012 | Randomized  Clinical trial | Single institution | EDV  Citicoline  CON | Within 24h  Within 24h  Within 24h | 4.0±0.53  3.91±1.13  4.2±0.7 | 14.27±7.47  15.58±11.36  16±9.23 | EDV: 30mg was given intravenously, twice daily;  Citicoline 1000mg every day;  Conventional treatment: comprised oxygen therapy, sedation, antiplatelet aggregation, and other treatment measures that decrease blood pressure and intracranial pressure, promote brain cell metabolism and blood circulation to remove blood stasis, and manage water and electrolyte imbalance. |
| Enomoto M^[32]^ | 2019 | Retrospective  study | Single institution | EDV  CON | Within 48h  Within 48h | 0-3  0-3 | NR  NR | EDV: 30mg was given intravenously, twice daily;  Conventional treatment: comprised oxygen therapy, sedation, antiplatelet aggregation, and other treatment measures that decrease blood pressure and intracranial pressure, promote brain cell metabolism and blood circulation to remove blood stasis, and manage water and electrolyte imbalance. |
| Alvarez-Sabín J^[13]^ | 2016 | Randomized  Clinical trial | Single institution | Citicoline  CON | Within 24h  Within 24h | NR  NR | 13  14 | Citicoline 1000mg every day;  Conventional treatment: comprised oxygen therapy, sedation, antiplatelet aggregation, and other treatment measures that decrease blood pressure and intracranial pressure, promote brain cell metabolism and blood circulation to remove blood stasis, and manage water and electrolyte imbalance. |
| Martynov MIu^[36]^ | 2012 | Retrospective  study | Single institution | Citicoline  CON | Within 24h  Within 24h | NR  NR | NR  NR | Citicoline 2000mg every day;  Conventional treatment: comprised oxygen therapy, sedation, antiplatelet aggregation, and other treatment measures that decrease blood pressure and intracranial pressure, promote brain cell metabolism and blood circulation to remove blood stasis, and manage water and electrolyte imbalance. |
| Clark WM^[16]^ | 1997 | Randomized  Clinical trial | Multicenter | Citicoline  CON | 14.63h  14.2h | 3.7  3.6 | 12.8  13.0 | Citicoline 500mg/ 1000mg/ 2000mg every day;  Conventional treatment: comprised oxygen therapy, sedation, antiplatelet aggregation, and other treatment measures that decrease blood pressure and intracranial pressure, promote brain cell metabolism and blood circulation to remove blood stasis, and manage water and electrolyte imbalance. |
| Li XX^[23]^ | 2020 | Randomized  Clinical trial | Single institution | EDV  CON | 2.13±0.19h  2.09±0.21h | NR  NR | ≥4  ≥4 | EDV: 30mg was given intravenously, twice daily;  Conventional treatment: comprised oxygen therapy, sedation, antiplatelet aggregation, and other treatment measures that decrease blood pressure and intracranial pressure, promote brain cell metabolism and blood circulation to remove blood stasis, and manage water and electrolyte imbalance. |
| Leon-Jimenez C^[35]^ | 2010 | Retrospective study | Multicenter | Citicoline  CON | Within 24h  Within 24h | NR  NR | 14.3  14.33 | Citicoline 2000mg every day;  Conventional treatment: comprised oxygen therapy, sedation, antiplatelet aggregation, and other treatment measures that decrease blood pressure and intracranial pressure, promote brain cell metabolism and blood circulation to remove blood stasis, and manage water and electrolyte imbalance. |
| Sun Z^[28]^ | 2019 | Randomized  Clinical trial | Single institution | EDV  CON | Within 24h  Within 24h | NR  NR | 22.20±8.03  22.25±9.01 | EDV: 30mg was given intravenously, twice daily;  Conventional treatment: comprised oxygen therapy, sedation, antiplatelet aggregation, and other treatment measures that decrease blood pressure and intracranial pressure, promote brain cell metabolism and blood circulation to remove blood stasis, and manage water and electrolyte imbalance. |
| Eiichi O^[31]^ | 2003 | Randomized  Clinical trial | Multicenter | EDV  CON | 37.3±22.6h  35.2±26.6h | NR  NR | NR  NR | EDV: 30mg was given intravenously, twice daily;  Conventional treatment: comprised oxygen therapy, sedation, antiplatelet aggregation, and other treatment measures that decrease blood pressure and intracranial pressure, promote brain cell metabolism and blood circulation to remove blood stasis, and manage water and electrolyte imbalance. |
| Ni J^[25]^ | 2020 | Randomized  Clinical trial | Single institution | Cinepazide maleate  CON | Within 48h  Within 48h | 0-5  0-5 | 7-10  7-10 | Cinepazide maleate: 320mg was given intravenously, once daily;  Conventional treatment: comprised oxygen therapy, sedation, antiplatelet aggregation, and other treatment measures that decrease blood pressure and intracranial pressure, promote brain cell metabolism and blood circulation to remove blood stasis, and manage water and electrolyte imbalance. |
| Tazaki Y^[29]^ | 1988 | Randomized  Clinical trial | Single intitution | Citicoline  CON | Within 24h  Within 24h | 0-5  0-5 | NR  NR | Citicoline 1000mg every day;  Conventional treatment: comprised oxygen therapy, sedation, antiplatelet aggregation, and other treatment measures that decrease blood pressure and intracranial pressure, promote brain cell metabolism and blood circulation to remove blood stasis, and manage water and electrolyte imbalance. |
| Mehta A^[39]^ | 2019 | Prospective  study | Single institution | Citicoline  EDV  Minoclycline  Cerebrolysin  CON | 13.47±4.34h  13.1±4.10h  12.40±3.87h  12.94±4.45h  13.30±4.95h | 4.5±0.17  3.75±0.19  4.2±0.21  4.3±0.20  4.45±0.15 | 14±4.34  13.25±5.3  13.1±4.59  14.15±5.30  13.35±4.53 | Citicoline 1000mg every day;  EDV: 30mg was given intravenously, twice daily;  Minoclycline: was administered as oral of 200 mg/d;  Cerebrolysin: 30ml was given intravenously;  Conventional treatment: comprised oxygen therapy, sedation, antiplatelet aggregation, and other treatment measures that decrease blood pressure and intracranial pressure, promote brain cell metabolism and blood circulation to remove blood stasis, and manage water and electrolyte imbalance. |
| Agarwal A^[7]^ | 2022 | Randomized  Clinical trial | Single institution | Citicoline  CON | Within 24h  Within 24h | NR  NR | 8-42  8-42 | Citicoline 2000mg every day;  Conventional treatment: comprised oxygen therapy, sedation, antiplatelet aggregation, and other treatment measures that decrease blood pressure and intracranial pressure, promote brain cell metabolism and blood circulation to remove blood stasis, and manage water and electrolyte imbalance. |
| Xu J^[5]^ | 2021 | Randomized  Clinical trial | Single institution | Edaravone dexborneol  EDV | Within 48h  Within 48h | NR  NR | 5-9  5-8 | Edaravone dexborned: 37.5mg was given intravenously, twice daily;  EDV: 30mg was given intravenously, twice daily |
| Xu J^[9]^ | 2019 | Randomized  Clinical trial | Multicenter | Edaravone dexborneol  EDV | Within 48h  Within 48h | 0-1  0-1 | 4-24  4-24 | Edaravone dexborned: 12.5mg, 37.5mg, or 62.5mg was given intravenously, twice daily;  EDV: 30mg was given intravenously, twice daily; |
| Zhu X^[38]^ | 2024 | Retrospective study | Single institution | Edaravone dexborneol  EDV | 3.27±1.13h  3.25±1.18h | NR  NR | 4-24  4-24 | Edaravone dexborned: 37.5mg was given intravenously, twice daily;  EDV: 30mg was given intravenously, twice daily |
| Dong Y^[19]^ | 2021 | Randomized Clinical trial | Multicenter | Ginkgolide  CON | Within 72h  Within 72h | NR  NR | 4-7  4-7 | Ginkgolide: 10ml was given intravenously daily;  Conventional treatment: comprised oxygen therapy, sedation, antiplatelet aggregation, and other treatment measures that decrease blood pressure and intracranial pressure, promote brain cell metabolism and blood circulation to remove blood stasis, and manage water and electrolyte imbalance. |
| Han X^[33]^ | 2023 | Retrospective study | Single  Institution | GDLM  CON | Within 48h  Within 48h | 0  0 | 3-6  3-7 | GDLM: 25mg was given intravenously daily;  Conventional treatment: comprised oxygen therapy, sedation, antiplatelet aggregation, and other treatment measures that decrease blood pressure and intracranial pressure, promote brain cell metabolism and blood circulation to remove blood stasis, and manage water and electrolyte imbalance. |
| Zhang X^[11]^ | 2021 | Randomized  Clinical trail | Multicenter | Ginkgolide  CON | Within 24h  Within 24h | NR  NR | 3-9  2-9 | Ginkgolide: 10ml was given intravenously daily;  Conventional treatment: comprised oxygen therapy, sedation, antiplatelet aggregation, and other treatment measures that decrease blood pressure and intracranial pressure, promote brain cell metabolism and blood circulation to remove blood stasis, and manage water and electrolyte imbalance. |
| Chen C^[14]^ | 2023 | Randomized  Clinical trial | Single institution | GDLM  CON | Within 24h  Within 24h | 2-3  2-3 | 8-11  9-11 | GDLM: 25mg was given intravenously daily;  Conventional treatment: comprised oxygen therapy, sedation, antiplatelet aggregation, and other treatment measures that decrease blood pressure and intracranial pressure, promote brain cell metabolism and blood circulation to remove blood stasis, and manage water and electrolyte imbalance. |
| Zhang Q^[10]^ | 2023 | Randomized  Clinical trial | Multicenter | GDLM  CON | Within 48h  Within 48h | NR  NR | 6-9  6-9 | GDLM: 25mg was given intravenously daily;  Conventional treatment: comprised oxygen therapy, sedation, antiplatelet aggregation, and other treatment measures that decrease blood pressure and intracranial pressure, promote brain cell metabolism and blood circulation to remove blood stasis, and manage water and electrolyte imbalance. |
| Hu X^[8]^ | 2023 | Retrospective study | Single institution | Edaravone dexborneol  CON | Within 48h  Within 48h | 3.9±1.3  4.0±1.2 | 10.0±6.1  10.6±6.4 | Edaravone dexborned: 37.5mg was given intravenously, twice daily;  Conventional treatment: comprised oxygen therapy, sedation, antiplatelet aggregation, and other treatment measures that decrease blood pressure and intracranial pressure, promote brain cell metabolism and blood circulation to remove blood stasis, and manage water and electrolyte imbalance. |

* EDV: edaravone; CON: conventional treatment; GDLM: ginkgo diterpene lactone meglumine; NR: Not report
