# Supplementary material for: Efficacy analysis of neuroprotective drugs in patients with acute ischemic stroke based on network meta-analysis
Source: Front Pharmacol. 2024 Nov 7;15:1475021. doi: 10.3389/fphar.2024.1475021 (PMC11578817; doi:10.3389/fphar.2024.1475021)
Supplement: Supplementary file 2 [file DataSheet1.doc]

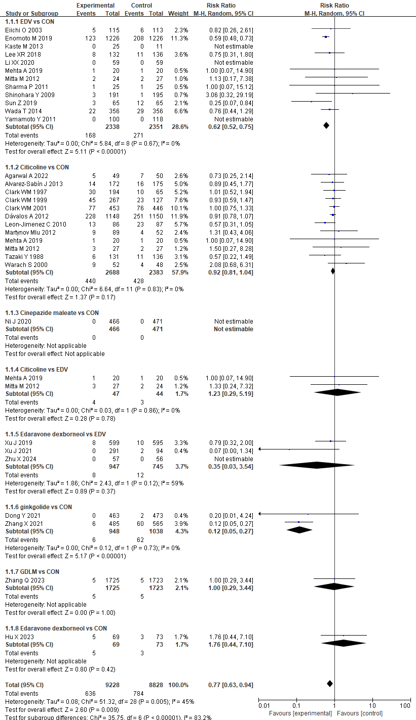


Supplementary Figure 1a. Subgroup analysis of the mortality of AIS


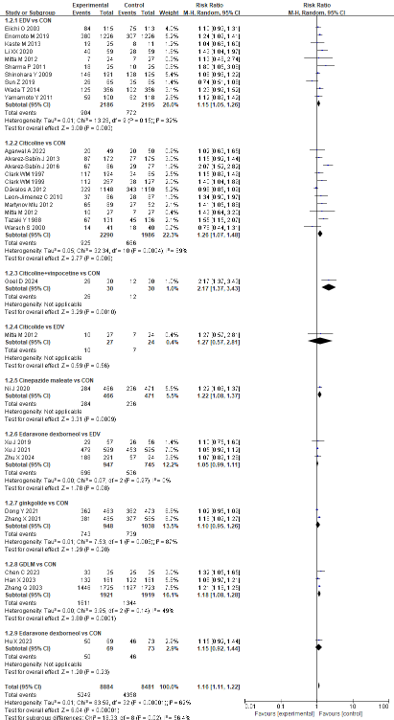


Supplementary Figure 1b. Subgroup analysis on patient proportion of the favorable result of AIS


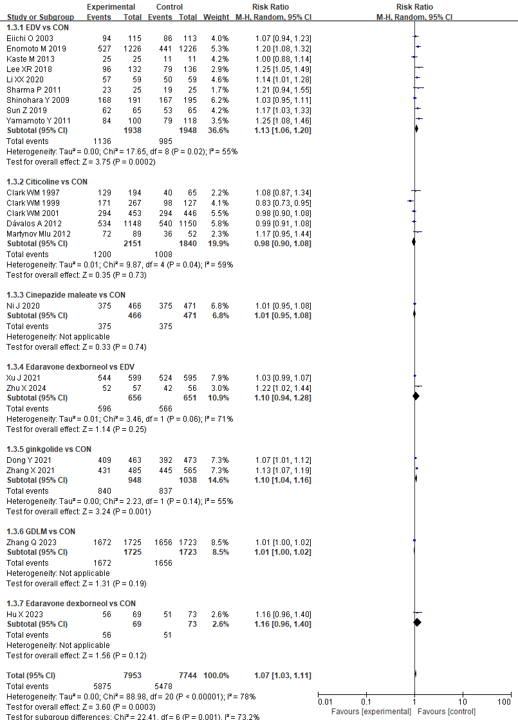


Supplementary Figure 1c. Subgroup analysis on patient proportion of the total treatment effective rate of AIS


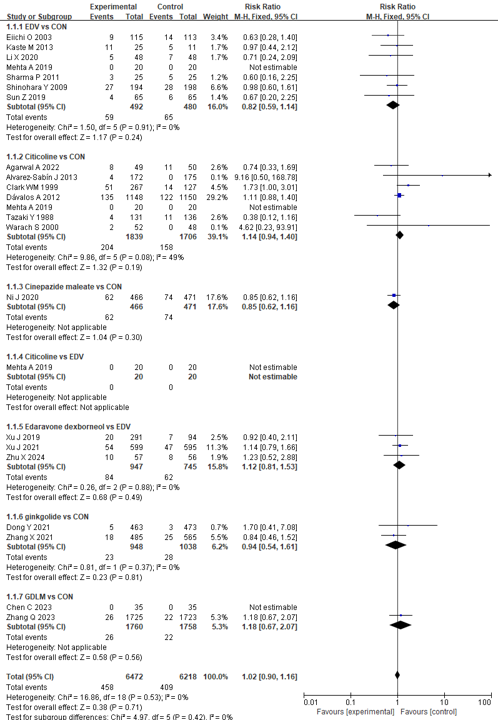


Supplementary Figure 1d. Subgroup analysis on the patient proportion of the adverse effect of AIS


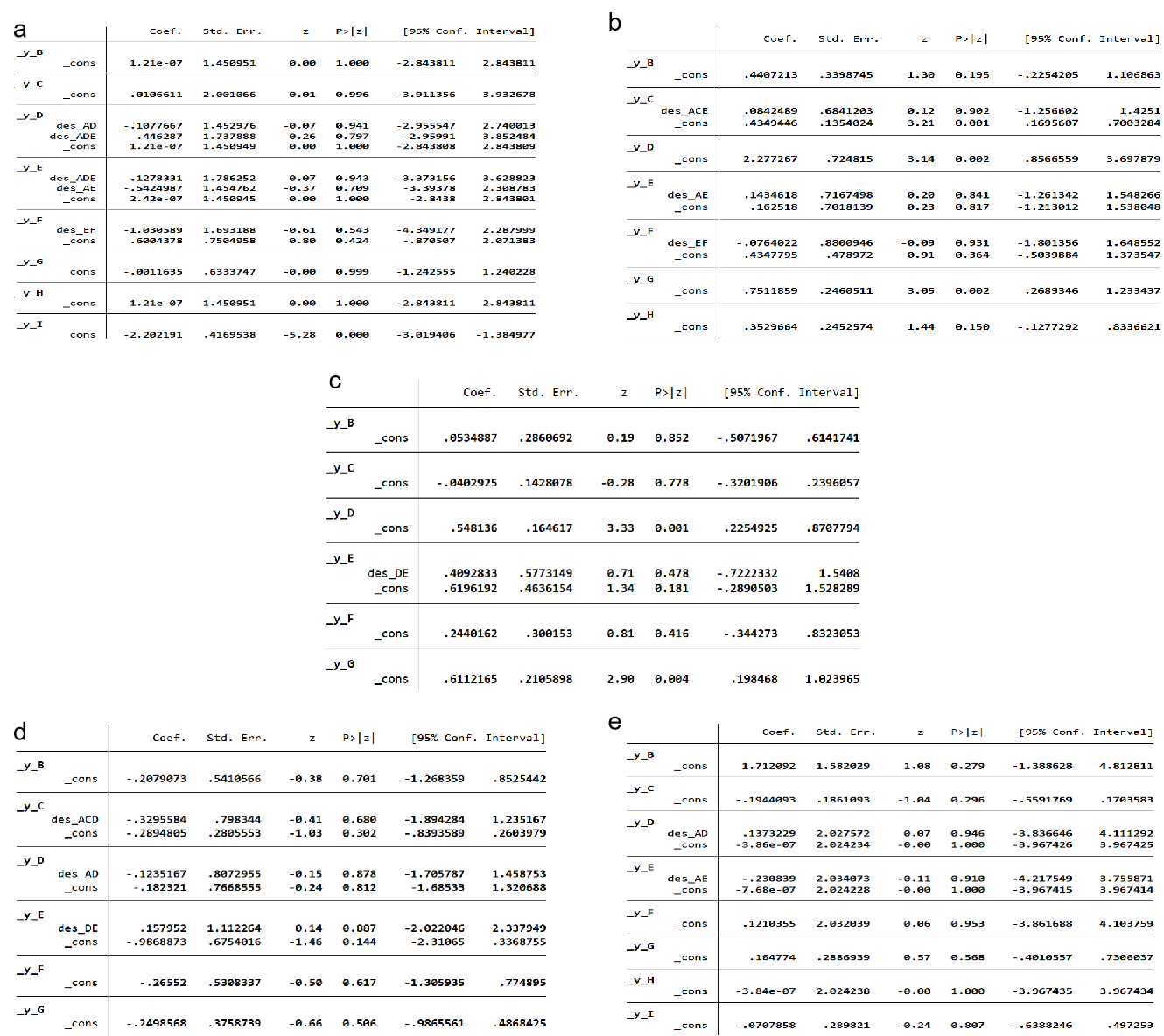


Supplementary Figure 2. The inconsistency test. The inconsistency test based on the mortality of AIS; B. The inconsistency test based on the patient proportion of the favorable result of AIS; C. The inconsistency test based on the patient proportion of the total treatment effective rate of AIS; D. The inconsistency test based on the patient proportion of the ineffective rate of AIS; E. The inconsistency test based on the patient proportion of the adverse effect of AIS
